# Prevalence and determinants of tobacco use amongst South African adults with mental illness in the Eastern Cape

**DOI:** 10.4102/sajpsychiatry.v27i0.1637

**Published:** 2021-06-14

**Authors:** Linda K. Tindimwebwa, Anthony I. Ajayi, Oladele V. Adeniyi

**Affiliations:** 1 Department of Psychiatry, Faculty of Health Sciences, Walter Sisulu University, East London, South Africa; 2 Cecilia Makiwane Hospital, East London, South Africa; 3 Population Dynamics and Sexual and Reproductive Health Research, African Population and Health Research Centre, Nairobi, Kenya; 4 Department of Family Medicine, Faculty of Health Sciences, Walter Sisulu University, East London, South Africa

**Keywords:** Eastern Cape province, mental health users, mental illness, South Africa, tobacco use

## Abstract

**Background:**

Given the physical and mental health consequences of tobacco use amongst individuals with mental illness, it was imperative to assess the burden of tobacco use in this population.

**Aim:**

This study examined the patterns and factors associated with tobacco use in individuals attending the outpatient unit.

**Setting:**

Cecilia Makiwane Hospital Mental Health Department in Eastern Cape province, South Africa.

**Methods:**

Lifetime (ever use) use and current use of any tobacco products were examined in a cross-sectional study of 390 individuals between March and June 2020. A logistic regression was fitted to determine the correlates of lifetime and current use of any tobacco products.

**Results:**

The rates of ever use and current use of tobacco products were 59.4% and 44.6%, respectively. Of the participants interviewed, lifetime tobacco use was more prevalent amongst individuals with schizophrenia (67.9%) and cannabis-induced disorders (97.3%) and lower in those with major depressive disorders (36.1%) and bipolar and related disorders (43.5%). Men were six times more likely to have ever used or currently use tobacco products in comparison to women. Also, those who had a salaried job or owned a business were over three times more likely to have ever used or currently use tobacco products compared with those receiving government social grants.

**Conclusions:**

The prevalence of tobacco use in this study was significantly higher than the general population in the Eastern Cape. Therefore, smoking prevention and cessation interventions targeted at the general population should target this often neglected sub-population in the region.

## Introduction

Tobacco use is a leading cause of morbidity and mortality worldwide, with an estimated 6 million deaths per year.^1^ Besides lung cancer, it is a significant risk factor for various respiratory illnesses, cardiovascular diseases and cerebrovascular accidents.^1^ According to the World Health Organisation (WHO), more than 1.1 billion people (20.2%) used tobacco globally.^2^ Of these, 66 million were in Africa, with a prevalence of 10.0%.

According to the National Department of Health, South Africa, tobacco use prevalence was 37% and 7% for men and women, respectively.^3^ However, there are variations in the prevalence of tobacco use according to gender, age and geographical location. Reddy et al.^4^ reported a prevalence of tobacco use of 17.6% amongst South African adults, with variations across provinces: Western Cape, Northern Cape, Eastern Cape and Free State had the highest prevalence at 32.9%, 31.2%, 31.4% and 27.4%, respectively. The study by Owolabi et al.,^5^ however, reported an even lower prevalence of 15% in Buffalo City Metropolitan Municipality (BCMM) in the Eastern Cape.^5^ However, the majority of the participants in their study were women. Globally, the low-income countries such as Haiti, Burkina Fasso and Bangladesh, respectively, reported current use of tobacco amongst the general population as 8.3%, 16.0% and 39.1%. In high-income nations such as Singapore, Canada, Seychelles and Austria, the figures reported were 16.5%, 17.5%, 21.1% and 29.1%, respectively (World Bank Group).^6^

Although several prevalence studies have been carried out on tobacco use in South Africa,^3,4,5^ it should be noted that these studies did not include individuals with mental illness. This identified gap in the overall understanding of tobacco use in this sub-population and the country needs to be addressed. Such data will help in designing user-specific interventions aimed at curbing tobacco use amongst individuals with mental illness.

Evidence suggests that individuals with long-standing mental illnesses are more likely to use tobacco products than the general population.^7^ Some studies have estimated up to a fivefold increase in the use of tobacco products in people with depression, anxiety, schizophrenia, eating disorders and attention deficit hyperactivity disorder (ADHD).^8,9,10,11^

Several theories have been advanced to explain the link between tobacco use and mental illness. There is also a bidirectional relationship between tobacco use and psychiatric symptoms.^12^ There is evidence of the association between tobacco use and changes in neural circuits and brain structures that are involved in psychiatric disorders.^8^ Individuals with mental illness tend to self-medicate with tobacco to ameliorate the symptoms.^13^ Conversely, studies have indicated that smoking itself may cause depression and anxiety.^14,15^

Tobacco use can also be a gateway drug to further substance use.^16^ Similarly, evidence suggests that second-hand smoke could lead to the development of generalised anxiety disorder, depression, conduct disorder and ADHD.^17,18^

Of particular importance in individuals with mental illness who use tobacco, particularly smokers, are the drug interactions. Some drugs (e.g. antidepressants, antipsychotics, mood stabilisers and sedative or hypnotics) used for treating mental illness are metabolised by hepatic cytochrome (CYP) 1A2 enzymes.^19^ Polycyclic aromatic hydrocarbons in cigarette smoke have been shown to induce CYP1A2 enzymes in heavy or moderate smokers. This enzyme induction impacts drug metabolism, leading to smokers requiring higher doses of medication to achieve therapeutic effects, and consequently increased risk of adverse effects.^10,20,21,22^

Given the physical and mental health consequences of tobacco use amongst individuals with mental illness, it is therefore imperative to assess the burden of tobacco use in this population. More so, most of the smoking cessation programmes tend to focus on the general population and, thus, neglect individuals with mental illness.^23^ There is little or no research on tobacco use in individuals with mental illness in South Africa, especially in the Eastern Cape province. This important data might guide the provincial health directorate in crafting user-specific interventions for this sub-population.

The objectives of this study were thus:

1. To describe the rate and pattern of tobacco use amongst individuals with mental illness attending the outpatient unit of a Mental Health Department in the Eastern Cape.
2. To examine the factors associated with tobacco use in the cohort.

## Methods

### Study design and setting

This cross-sectional study was conducted between March and June 2020 at the outpatient unit of a Mental Health Department in the Eastern Cape. This department is affiliated with a large university for the training of both undergraduate and postgraduate students in the Eastern Cape. It offers mental healthcare services to the entire BCMM and Amathole district in the Eastern Cape province, which has a combined population of 1 674 637 people living in it.^23^ It comprises inpatient and outpatient units of 25 beds each. The outpatient unit sees between 13 000 and 15 000 patients per year. The facility users reflect the racial distribution of the entire BCMM: black people (85.1%), white people (7.7%), mixed-race people (6%), Indians (0.8%) and others (0.3%), and the predominant language spoken is isiXhosa.^24^

### Participants and sample size

Using Cochran’s formula^25^ for cross-sectional study at a confidence level of 95%, a precision level of ± 5%, tobacco use prevalence of 50% and anticipated missing responses of 5%, the sample size was estimated as 390.

Sample Size (*N*) = *Z*_1-_α_/2_^2^*p*(1−*p*)/*d*^2^

where *z*_1-_α_/2_ is the standard normal variate at 1.96.

*P* is the expected proportion of patients with dual diagnosis based on previous studies,^26^ and observation of patients admitted in the wards at Cecilia Makiwane Hospital Mental Health Unit (CMHMHU), at 50%, which is equal to 0.5.

*d* = 0.05

Sample size (*N*) = 384.16, which was increased to 390.

Individuals attending the outpatient unit of the Mental Health Department were considered eligible for the study. Participants were included if they were 18 years and above, had been on treatment for the underlying mental illness for at least 1 year and were clinically stable to be interviewed for the study. Based on history taking and mental state examination, the attending doctors on each day carried out a risk assessment on patients. Individuals who posed a threat to themselves, others and/or property were managed according to the protocol of the department and were excluded from the study. Eligible participants were recruited consecutively after being in consultation with the attending doctor each day. The attending doctor administered the questionnaire and also reviewed the medical records of the participants.

### Study instrument

A structured questionnaire comprising three sections was compiled. The sections were as follows: demographic data, use of tobacco products and other substances and the current diagnosis of mental illness, using categories from the Diagnostic and Statistical Manual of Mental Disorders, 5th Edition (DSM-5).^27^

### Validity and reliability

To ensure the construct and face validity of the study instrument, relevant items from the pre-validated WHO STEPwise^28^ and National Institute on Drug Abuse (NIDA)^29^ questionnaires were selected and collated.

Although some of these validated questionniares have been used successfully in South Africa,^5^ we further tested the validity and reliability of the adapted instrument in this special population through a pilot study with 10 patients in the setting. Adjustments were made using clinician feedback and participant responses.

Specifically, we realised from the pilot study that only medical doctors (excluding intern doctors) were needed for risk assesment and recruitment of stable patients for this study. The current use of cigarette smoking was defined as smoking in the past 12 months. However, the pilot study’s results were excluded from the final analysis.

### Study procedure

Group information detailing the purpose and process of the study was provided to patients by the principal investigator before their consultations. Doctors were trained by the principal investigator on the selection criteria and how to administer the questionnaire after consultation with the participants. The main doctor in the outpatient clinic for each day administered the questionnaire (after the consultation) and also reviewed the clinical notes for additional information. Given that this study was carried out during the first wave of coronavirus disease 2019 (COVID-19) with the nation’s lockdown, the data collection lasted for 4 months – from March 2020 to June 2020 – and was mostly carried out during weekdays.

### Main outcome measure

Tobacco use was defined as the use of any products: cigarettes, cigars, loose tobacco rolled in paper or smoked in a pipe, chewable tobacco and inhaled snuff. Evidence suggests that there is no safe limit for smoking in any individual.^30,31^ Hence, any form or amount of tobacco use is considered an unhealthy lifestyle behaviour. This was categorised as self-reported current use (in the previous year) and ever use of any tobacco products. In addition to self-reported tobacco usage, clinical notes of each participant were reviewed to validate the responses given. Where there were discrepancies, the participants’ clinical notes were taken as the true status of tobacco use.

### Covariates

Relevant demographic characteristics such as age, sex, race, level of education, marital status, employment, monthly income and area of residence were obtained through interviews. Demographic characteristics were categorised according to a similar report from the general population in the same setting.^5^ Participants’ ages (in years) were categorised as follows: 18–25, 26–35, 36–45, 46–55, 56–65 and 66 years or older. Socio-economic status followed the pattern described by Geyer et al.^32^: level of education, occupation and income. The highest level of education attained by the participants was categorised as follows: none, grades 1–7, grades 8–12 and tertiary.

Household monthly income in rands was categorised as follows: none, R150–R2000, R2001–R5000 and R5001 or more. Employment status was categorised as none, having a job or business and social assistance (disability grant, child grant, pension). Participants were considered unemployed if they were unemployed in the formal or informal sector.

### Statistical analysis

Data were entered into an Excel spreadsheet and double-checked for accuracy. Analysis was conducted using the IBM Statistical Package for Social Sciences Version 24.0 for Windows (IBM Corp. Armonk, New York, the United States of America). Categorical variables were reported as frequencies (*n*) and percentages (%), whilst continuous variables were reported as means (± standard deviation). Simple descriptive statistics were used to summarise the socio-demographic characteristics of the participants. Bivariate analysis was conducted to examine the associations between background characteristics and tobacco use. Multivariate logistic regression analysis was used to examine the correlates of tobacco use. A *p*-value of < 0.05 was considered to be statistically significant.

### Ethical considerations

Ethical approval was obtained from the Research Ethics Committee of the affiliated university in the Eastern Cape (Reference number 093/2019). Permission was obtained from the Eastern Cape Department of Health, and the clinical governance of the research site. An information sheet (written in English and Xhosa) detailing the purpose and procedure of the study was given to each participant. Each participant or legal guardian (if the participant was unable to consent because of diminished capacity in individuals with neurocognitive disoders and intellectual impairment) gave written informed consent for voluntary participation in the study. This decision was made by the attending doctor. The study followed the Helsinki Declaration on human and animal research guidelines. Participants’ rights to privacy, confidentiality and anonymity were respected during and after the study. Unique code identifiers were assigned to each participant in order to maintain their anonymity. All hard copies of materials used in the study were locked in a protected cabinet, whilst soft copies were password protected.

## Results

### Socio-demographic characteristics of the study participants

Most of the participants were men (59.5%), black (87.2%) and single (74.9%); had an education level of grades 8–12 (57.2%); and were between 46 and 55 years of age (27.2%). In addition, 81% were unemployed, 67.4% had social assistance (disability grants, pensions and child-care grants) as their source of income and 46.9% had a combined household income of R2000–R5001 per month. The majority of participants lived in urban areas (83.6%) with their families (84.9%) (Table 1).

**TABLE 1:** Socio-demographic characteristics of the study participants.

Variables	Frequency	%
**Gender**		
Male	232	59.5
Female	158	40.5
**Age (years)**		
18–25	37	9.5
26–35	87	22.3
36–45	90	23.1
46–55	106	27.2
56–65	48	12.3
66 and above	22	5.6
**Race**		
Black people	340	87.2
White people	34	8.7
Mixed race people	14	3.6
Indian or Asian people	2	0.5
**Education**		
No formal education	13	3.3
Grades 1–7	89	22.8
Grades 8–12	223	57.2
Tertiary	65	16.7
**Marital status**		
Single	292	74.9
Married	37	9.5
Divorced or separated	25	6.4
Widowed	20	5.1
Cohabiting	16	4.1
**Employment**		
Employed	33	8.5
Unemployed	316	81.0
Student	11	2.8
Retired	30	7.7
**Household income (in rand)**		
None	24	6.2
150–2000	114	29.2
2001–5000	183	46.9
5001 R and over	69	17.7
**Source of income**		
None	94	24.1
Job or business	33	8.5
Social assistance	263	67.4
**Place of residence**		
Rural	64	16.4
Urban	326	83.6
**Living arrangement**		
Living alone	19	4.9
Living with friends	18	4.6
Living with family (nuclear or extended)	331	84.9
Living with partner or spouse only	22	5.6

### Tobacco use

Of the participants interviewed (*N* = 390), 54.9% reported having ever used and 44.6% reported current use of tobacco products. In the bivariate analysis, age, gender and source of income were significantly associated with having ever used and current use of tobacco products (*p* < 0.05) (Table 2).

**TABLE 2:** Association between background characteristics and tobacco use.

Variables	Ever used tobacco products			Tobacco use in the previous year		
	Yes		*p*	Yes		*p*
	*n*	%		*n*	%	
All	214	54.9		174	44.6	
**Gender**						
Male	165	71.1	< 0.001	141	60.8	< 0.001
Female	49	31.0		33	20.9	
**Age (year)**						
18–35	71	57.3	0.025	59	47.6	0.002
36–45	54	60.0		46	51.1	
46–55	62	58.5		52	49.1	
56 and above	27	38.6		17	24.3	
**Education**						
No formal education and up to Grade 7	53	52.0	0.091	46	45.1	0.147
Grades 8–12	132	59.2		106	47.5	
Tertiary	29	44.6		22	33.8	
**Marital status**						
Single	182	54.0	0.237	145	43.0	0.075
Married or cohabiting	32	60.4		29	54.7	
**Employment**						
Employed	22	66.7	0.096	17	51.5	0.096
Unemployed	176	55.7		145	45.9	
Student	5	45.5		5	45.5	
Retired	11	36.7		7	23.3	
**Household income (in rand)**						
0–2000	82	59.4	0.205	66	47.8	0.580
2001–5000	100	54.6		80	43.7	
5001 and over	32	46.4		28	40.6	
**Source of income**						
None	64	68.1	0.001	50	53.2	0.025
Job or business	23	69.7		19	57.6	
Social assistance	127	48.3		105	39.5	
**Place of residence**						
Rural	33	51.6	0.328	28	43.8	0.495
Urban	181	55.5		146	44.8	
**Living arrangement**						
Living alone	13	68.4	0.303	9	47.4	0.554
Living with friends	12	66.7		8	44.4	
Living with family (nuclear and/or extended)	175	52.9		144	43.5	
Living with partner or spouse only	14	63.6		13	59.1	

Of the participants interviewed (*N* = 390), lifetime tobacco use was more prevalent amongst individuals with schizophrenia (67.9%) and cannabis-induced disorders (97.3%) and was lower in those with major depressive disorders (36.1%) and bipolar and related disorders (43.5%).

Current tobacco use was higher amongst individuals with schizophrenia (58.5%) and cannabis-induced disorders (86.7%) and was lower amongst those with major depressive disorder (49.1%) and bipolar and related disorders (27.5%). The difference was statistically significant (*p* < 0.05) (Table 3).

**TABLE 3:** Association between common mental illnesses and tobacco use.

Variables	Life time tobacco use		Previous year use	
	*n*	%	*n*	%
**Major depressive symptoms**				
Yes	26	36.1	18	25.0
No	188	59.12**	156	49.1**
**Bipolar related**				
Yes	30	43.5	19	27.5
No	184	57.3*	155	48.3*
**Schizophrenia**				
Yes	108	67.9**	93	58.5**
No	106	45.9	81	35.1
**Other psychotic disorders**				
Yes	10	38.5	9	34.6
No	204	56.0	165	45.3
**Alcohol-related disorders**				
Yes	26	66.7	22	56.4
No	188	53.6	152	43.3
**Cannabis-related disorders**				
Yes	73	97.3*	65	86.7**
No	141	44.8	109	34.6

* , *p* > 0.05;

** , *p* > 0.001.

### Multivariable findings

In the unadjusted (logistic regression model) analysis, male gender, younger age, having grade 8–12 level of education and source of income were significantly associated with a higher likelihood of having ever used tobacco products. However, after adjusting for relevant covariates, only male gender, being unemployed and having a salaried job or owning a business were significantly associated with a higher odds of having ever used tobacco products. Men were six times more likely to have ever used tobacco products in comparison to women. Also, those who had a salaried job or owned a business were over three times more likely to have ever used tobacco products compared with those receiving government social grants.

Similar findings emerged regarding tobacco use in the previous year (current use). Male gender, single marital status, being employed and aged 36–55 years were significantly associated with current use of tobacco products in the unadjusted model. In the adjusted model, men were six times more likely to have used any tobacco products in the previous year compared with women. Individuals aged 36–55 years were twice more likely to have used tobacco products in the previous year compared with those aged 56 years and above, after adjusting for other covariates. Also, single individuals were 58% less likely to have used any tobacco products in the previous year compared with married individuals. Those who had a salaried job or owned a business were about three times more likely to have used tobacco products compared with those on social grants (Table 4).

**TABLE 4:** Multiple logistic regression analysis showing predictors of tobacco use.

Variables	Ever used tobacco products				Previous year use of tobacco products			
	Unadjusted		Adjusted		Unadjusted		Adjusted	
	OR	95% CI	OR	95% CI	OR	95% CI	OR	95% CI
**Gender**								
Male	5.48	3.53–8.51**	5.67	3.49–9.27**	5.87	3.69–9.35**	6.14	3.66–10.31**
Female (ref)	1	-	1	-	1	-	1	-
**Age (year)**								
18–35	2.13	1.17–3.88*	1.09	0.54–2.21	2.83	1.48–5.42*	1.78	0.85–3.72
36–45	2.39	1.26–4.53*	1.61	0.78–3.31	3.26	1.64–6.47*	2.42	1.13–5.16*
46–55	2.24	1.21–4.16*	1.68	0.84–3.36	3.00	1.54–5.84*	2.24	1.07–4.67*
56 and above (ref)	1	-	1	-	1	-	1	-
**Education**								
No formal education and up to Grade 7	1.34	0.72–2.51	1.20	0.57–2.54	1.61	0.84–3.06	1.49	0.70–3.17
Grades 8–12	1.80	1.03–3.14*	1.61	0.84–3.11	1.77	0.99–3.15	1.53	0.78–2.98
Tertiary (ref)	1	-	1	-	1	-	1	-
**Marital status**								
Single	0.77	0.43–1.39	0.66	0.33–1.31	0.63	0.35–1.12	0.42	0.21–0.85*
Married or co-habiting (ref)	1	-	1	-	1	-	1	-
**Source of income**								
Unemployed	2.29	1.39–3.75*	2.17	1.22–3.85*	1.71	1.06–2.75*	1.49	0.86–2.59
Job or business	2.46	1.13–5.38*	3.45	1.37–8.67*	2.04	0.98–4.25	2.51	1.04–6.05*
Social assistance (ref)	1	-	1	-	1	-	1	-
**Place of residence**								
Rural	0.85	0.50–1.46	0.77	0.42–1.41	0.96	0.56–1.65	0.86	0.47–1.57
Urban (ref)	1	-	1	-	1	-	1	-

OR, odds ratio; ref, reference category; CI, confidence interval.

* , *p* > 0.05;

** , *p* > 0.001.

## Discussion

Previous studies on tobacco use in South Africa focused on the general population without people with underlying mental illnesses,^3,4,5^ thus creating a gap in the overall understanding of tobacco use in the country.

As such, interventions aimed at tobacco use cessation tend to neglect individuals with mental illness. This study examined the prevalence of tobacco use (lifetime and current use) of individuals with mental illness in the Eastern Cape province. Tobacco use increases the risk of mental illnesses and directly interferes with the metabolism of certain psychotropic drugs,^8,19^ and therefore it deserves attention.

This study found a high prevalence of lifetime (54.9%) and current tobacco use (44.6%) amongst individuals with mental illness in the study cohort. These rates are higher than those found in the general population in the region (15%)^5^ and the country (17.6%).^4^ This finding corroborates other reports elsewhere in the world. In the United States of America, adults with mental illness smoked more in the previous month (33.3%) than those without a mental illness (20.7%).^33^ A similar pattern was reported in Brazil, with a current tobacco use prevalence of 53% in individuals with mental illness in comparison to 18% in the general population.^34^ Evidence suggests that despite a reduction in tobacco use in the general population in countries such as the United States of America, the United Kingdom and Australia, the prevalence of smoking amongst people with mental illness is two to three times that of the general population.^10,35,36^

Of interest in this population is the historical context of tobacco use within mental health facilities.

Tobacco products were used as rewards to reinforce desirable behaviours and in substance rehabilitation centres as an acceptable substitute for other substances.^37^ This may have further added to the burden created by tobacco use in an environment where individuals should be assisted with tobacco cessation. Most mental health facilities no longer permit smoking indoors, but some still operate partial smoke-free policies that allow inpatients to smoke outdoors.^38^ The movement towards the improvement of the health of individuals with mental illness by the establishment of completely smoke-free mental health facilities has gained traction over the years.^39^

The present study found a significant association between patterns of tobacco use and the underlying mental illnesses in the cohort. Higher proportions of individuals with schizophrenia (67.9% and 58.5%) and cannabis-related disorders (97.3% and 86.7%) reported lifetime and current tobacco use, respectively, in this study. However, fewer individuals with bipolar disorders (43.5% and 27.5%) and major depressive disorders (36.1% and 25%) reported lifetime and current tobacco use, with the difference being statistically significant.

These findings corroborate a previous report in the United States of America that found between 70% and 85% of individuals with schizophrenia smoked in comparison to 50% – 70% with bipolar disorder.^40^ The National Institutes of Health panel observed that smoking prevalence was the highest amongst individuals with scizhophrenia (90%) and that nicotine dependence was especially challenging for this group.^41^ Smokers with schizophrenia smoke more cigarettes and are more nicotine-dependent than the general population,^42,43,44^ and additionally had the highest rates of smoking in comparison with other mental illnesses.^39^ Despite the overwhelming evidence on the relationship between smoking and mental illness, there is a lack of tobacco cessation programmes targeting individuals with mental illness. This consequently may result in poorer health outcomes for the patients who are at increased risk of metabolic side effects of antipsychotics and mood stabilising drugs, in addition to the significant risk from tobacco use.

In this study, male individuals were six times more likely to report lifetime or current tobacco use than their female counterparts. This is similar to the findings in the same setting by Owolabi et al.^5^ albeit in the general population. Studies carried out in sub-Saharan Africa, the United States of America and Europe have also shown similar trends.^34,45,46^ However, reports from Sweden and Iceland showed equal use of tobacco products in both genders.^46^ The higher prevalence of men who use tobacco in this study and in South Africa in general can be ascribed to cultural and societal views where tobacco use is more acceptable amongst men than among women.^47^

Close relationships such as marriage or cohabitation have been shown to be beneficial in relation to lifestyle behaviours.^48^ Findings of this study showed that married or cohabiting people were twice as likely to smoke in comparison to single individuals. This is a surprising result, given that the opposite is true in the same setting amongst the general population.^5^ Several other studies have also reported contrasting results to the current study.^49,50^ However, all of these studies did not include individuals with mental illness. It is plausible in this population that a lack of insight, which is a feature of mental illness, may over-ride the protection offered by an intimate relationship. More studies of individuals with mental illness in the country will provide clarity on this association.

This study showed that individuals who were employed have higher odds of current tobacco use compared with those on government social grants. This result should be treated with caution, given the small number of individuals who were employed (8.5%) in comparison to those unemployed (81%). However, these results followed a similar pattern to the rest of the general population in the same setting. Owolabi et al. reported a prevalence of 21% and 14.1% amongst employed and unemployed adults, respectively, in the region.^5^

The prevalence of tobacco use in the present study is far higher for each category of tobacco use: 66.7% and 51.5% for lifetime use and current use amongst employed individuals in comparison with 55.7% and 45.9% amongst unemployed individuals with mental illness. Evidence points to an association between smoking and unemployment^51^; however, it is unclear whether smoking is a cause or effect of unemployment.^52^ Many studies from Europe and New Zealand have reported contrasting results that showed higher rates of smoking in unemployed compared to employed individuals in the general population.^53,54,55^ In addition, Ham et al. found that the type of work in itself is a determinant of tobacco use; specifically, the blue-collar workers were more likely to start smoking cigarettes at a younger age and heavily than white-collar workers.^56^

In this study, individuals between the ages of 35 and 55 years were more likely to use tobacco in comparison to other age categories. A similar trend was reported by Owolabi et al., with the highest rate of smoking (19.2%) observed between 46 and 55-year-olds in the general population.^5^ Champagne et al. reported a similar trend in Latin America, where the highest prevalence was in the age group of 35–44 years.^57^ This age group represents a significant percentage of working people. Therefore, it can be deduced that access to money made a difference in the pattern of tobacco use in this population.

## Strength and limitations

Although this study was conducted in a single health facility centre, it should be noted that this hospital offers mental healthcare services to the entire BCMM and Amathole district of the Eastern Cape province, which has a combined population of 1 674 637 people living in the region.^24^ As such, this study highlights a very important public health issue in an often-neglected population. Findings of this study may be useful for health authorities and health in planning on how to improve the overall well-being of people with mental health problems in the region.

The cross-sectional design and the self-reporting of the main outcome measures are notable limitations of the study. As a result of the cross-sectional nature of this study, causal inference cannot be drawn. Also, if the attending doctor is administering the questionnaire, it could lead to reporting bias on tobacco use. However, this was mitigated by cross-checking the clinical notes and where there were discrepancies, the information in the clinical note was recorded in the questionnaire. Future studies in this population should assess nicotine dependence and further build on findings of the present study.

## Conclusion

We found a high prevalence of tobacco use in the study sample, particularly amongst individuals who were married or in cohabiting relationship, employed and aged between 36 and 55 years. In addition, individuals with underlying schizophrenia and cannabis-related disorders were at higher risk of lifetime or current use of tobacco products. Therefore, mental health clinicians should design a screening tool for tobacco use in this population with a view to identify individuals for cessation interventions. Smoking cessation interventions for people with mental illness require targeted research in the country.
